# Number of remaining teeth and frailty in community-dwelling older adults: an exploratory analysis of the potential mediating role of oral health-related quality of life

**DOI:** 10.3389/froh.2026.1927873

**Published:** 2026-10-08

**Authors:** Dongxin Da, Suyu Ge, Qingqing Weng, Hao Zhang, Xiaoli Zeng, Yiwei Jiang, Jin Yu, Huning Wang, Ying Zhang

**Affiliations:** 1 Department of Preventive Dentistry, Shanghai Stomatological Hospital and School of Stomatology, Fudan University, Shanghai, China; 2 Shanghai Key Laboratory of Craniomaxillofacial Development and Diseases, Fudan University, Shanghai, China

**Keywords:** dental health survey, frailty, mediation analysis, OHIP-14, remaining teeth

## Abstract

**Objectives:**

Previous studies have indicated associations between the number of remaining teeth, oral health-related quality of life (OHRQoL), and frailty. We aimed to investigate the association between number of remaining natural teeth and frailty among Chinese community-dwelling older adults in Shanghai, and to explore whether OHRQoL mediates this association.

**Methods:**

In this cross-sectional study, 1,122 Chinese community-dwelling older adults in Huangpu District, Shanghai, underwent oral examinations and completed questionnaires. Oral examinations were conducted according to the World Health Organization's Basic Methods for Oral Health Surveys (5th edition). Frailty was assessed using the FRAIL scale, and OHRQoL was measured with the Chinese version of Oral Health Impact Profile-14 (OHIP-14). A counterfactual-based causal mediation analysis with 5,000 bootstrap resamples was performed, using the three-category frailty outcome (robust/pre-frail/frail), to evaluate the mediating role of OHRQoL in the association between number of remaining teeth and frailty.

**Results:**

Among the 1,122 participants, 225 were diagnosed with frailty, accounting for 20.05% of the participants. Age, sex, education, body mass index, and number of remaining teeth significantly differed between participants with and without frailty (*P* < 0.01). After adjusting the covariates, logistic regression models showed that OHRQoL was significantly associated with frailty, with odds ratio (OR) = 1.140 [95% confidence interval (CI) 1.109–1.171]. In an exploratory analysis, OHRQoL was statistically consistent with a potential partial mediating role (proportion mediated = 19.3%, 95% CI 11.6%–31.2%).

**Conclusions:**

There is a potential association between number of remaining teeth and frailty, and OHRQoL may play a partial mediating role in this relationship. Given the cross-sectional design, causal inference requires longitudinal confirmation. Oral health—particularly, the number of remaining teeth—should be a key focus for both the elderly and healthcare providers.

## 1 Introduction

Population aging is accelerating globally. In 2020, the global population aged 60 and above reached 1 billion, accounting for 13.4% of the world's population. The number of adults aged 80 years or older is expected to triple between 2020 and 2050 to reach 426 million (1). In China, according to the National Health Commission, by the end of 2022, the elderly population aged 60 and above reached 280.04 million, accounting for 19.8% of the Chinese population (2). It is expected that by around 2035, the elderly population aged 60 and above will exceed 400 million, accounting for more than 30% (3). The prevalence of oral diseases in the elderly in China is over 98%, with the prevalence of caries being over 95% and the prevalence of healthy periodontium being less than 10% (4). In 2019, oral diseases accounted for 2.76 million years of healthy life lost due to disability (YLD) and 4.32 million disability-adjusted life years (DALY), significantly reducing life expectancy. Oral health-related direct economic expenditure reached more than 400 billion Chinese Yuan, imposing a heavy economic burden (5, 6).

Aging is characterized by geriatric syndromes, which arise due to multiple underlying factors, such as frailty. Frailty is characterized by progressive decline in physical, cognitive, and social functioning. Frail people are prone to falls, infections, hospitalizations, and death, with risk influenced by malnutrition, chronic disease, physical inactivity, and social isolation. Studies have shown that frailty not only significantly affects the quality of life but also increases the medical burden (7, 8). It is important to identify influencing factors, preventive strategies, and intervention measures to improve the health status of the older adults. However, due to the unclear risk factors and pathogenesis of frailty, further research is needed.

Oral diseases, as a risk factor for frailty, directly affect one's nutritional intake, verbal communication, and overall health (9–11). Oral diseases include a range of conditions affecting the mouth, teeth, gums, and surrounding tissues, such as dental caries, periodontal diseases, oral infections, and tooth loss. Tooth loss is a common oral disease in the elderly, affecting chewing function, and is also associated with psychological problems such as malnutrition, social isolation, and depression. There may be a correlation between oral health and frailty (12–14), but there is a lack of research on the mediators between tooth loss and frailty.

Previous studies have found that tooth loss, denture use, and periodontitis may be related to the development of frailty (13, 15, 16). However, most studies have focused on clinical oral conditions (e.g., tooth loss, periodontitis) rather than subjective oral health-related quality of life (OHRQoL). Furthermore, few studies have explored potential mechanistic pathways of the mediators, particularly in urban community-dwelling older Chinese adults.

Based on the existing evidence, we hypothesized that (1) a lower number of remaining teeth is associated with a higher risk of frailty and (2) this association is partially explained by poorer OHRQoL. Tooth loss may compromise mastication, comfort, oral function, and social interaction, which in turn contribute to frailty through nutritional, psychological, and functional pathways (number of remaining teeth → OHRQoL → frailty). We tested these hypotheses in a cross-sectional sample of 1,122 community-dwelling older adults.

## 2 Methods

### 2.1 Study population

From September 2020 to October 2021, we conducted a cross-sectional study of community-dwelling older adults in the Bund Street subdistrict, Huangpu District, Shanghai, China. The sampling frame comprised 2,421 registered residents aged 60 years and older. From this register, 1,210 residents were selected by a 1:2 sampling scheme (one in every two registered residents); of these, 1,122 (92.7%) provided written informed consent and completed the questionnaire survey and oral examination, while the remaining 88 (7.3%) refused to participate, did not meet the eligibility criteria, or had incomplete questionnaire data. This study was conducted in accordance with the Declaration of Helsinki and its later amendments.

Inclusion criteria were (1) ability to perform basic self-care; (2) ability to communicate fluently and independently; and (3) provision of informed consent and willingness to participate in the survey. Exclusion criteria were (1) history of severe head trauma, brain disease, or mental disorder; (2) unable to complete the full survey; and (3) considered unsuitable for participation.

### 2.2 Oral health evaluation

The number of remaining teeth was self-reported by the participants and confirmed by the investigators through oral examinations. Remaining teeth were quantified as the total number of permanent teeth in adults, including the third molars. Edentulism was defined as complete loss of all natural teeth. Participants completed the Oral Health Impact Profile (OHIP-14) scale under interviewer guidance. The OHIP-14 scale is the primary scale used to assess OHRQoL. OHIP-14 was developed by Slade based on Locker's oral health model and was further simplified into a 14-item scale in 1997 (17). In 2006, Xin and Ling translated the English version of OHIP-14 into Chinese and validated its reliability and validity (18). The OHIP-14 consists of 14 questions and seven dimensions: functional limitation, physical pain, psychological discomfort, physical disability, psychological disability, social disability, and handicap (18). Each question has five options, using a Likert scale ranging from 0 to 4. The total score for the 14 questions ranges from 0 to 56, with higher scores indicating poorer oral health-related quality of life (Supplementary Material).

### 2.3 Frailty assessment

The FRAIL scale was used to assess frailty among the participants, originally proposed by the Geriatric Advisory Panel in 2008 (19). In 2017, Yin et al. translated the scale into Chinese and verified its reliability and validity (20). The FRAIL scale comprises the following five domains: fatigue, resistance (defined as the ability to climb stairs), ambulation (ability to walk a certain number of meters), number of illnesses, and loss of weight (>5%). In this study, a score of 0 was classified as robust, scores of 1 or 2 were categorized as pre-frail, and scores of 3, 4, or 5 were defined as frail (Supplementary Material).

### 2.4 Data collection of other variables

Trained researchers conducted face-to-face interviews with the participants to collect demographic characteristics (age, sex, height, weight, years of education, etc.), lifestyle habits (smoking and alcohol drinking for at least 1 year), and medical histories including diabetes mellitus and hypertension. The type of dental prosthesis worn was recorded as no denture, fixed crown/bridge, or removable complete/partial denture (Supplementary Material). Body mass index (BMI) was calculated as weight in kilograms divided by the square of height in meters. Histories of chronic diseases were verified for consistency by cross-checking medical records.

### 2.5 Sample size calculation

According to the preliminary survey, approximately 20% of elderly adults in Shanghai's communities suffered from frailty, with a permissible error margin of about 3%. As this is a cross-sectional study, participants were drawn from the resident register using a 1:2 sampling scheme. Based on sample size calculation formula, the required sample was is at least 1,024 participants. Allowing for a 5% non-response and invalid questionnaire rate, the minimum sample size was 1,078 participants. The sample size was originally calculated to estimate frailty prevalence in this cross-sectional survey rather than specifically for the association or mediation analyses. Nonetheless, the achieved sample (*n* = 1,122, including 225 frail participants) provides adequate statistical power for the associational and mediation analyses, given the observed effect sizes [e.g., odds ratio (OR) ≈ 0.92 per additional remaining tooth, equivalently OR ≈ 1.08 per missing tooth]. Thus, the current sample size was sufficient:

$$n=(Z_{1-(\alpha /2)}/\delta {)}^{2}\times p\times (1-p)$$

### 2.6 Statistical analysis

Continuous variables were described as mean (standard deviation, SD) or median (interquartile range, IQR), as appropriate, and frequencies (%) were used for categorical variables. For the three-group comparisons in Table 1, one-way analysis of variance (ANOVA) was used for continuous variables, and the Pearson chi-squared test was used for categorical variables. The associations of OHRQoL and the number of remaining teeth with frailty were examined using multinomial logistic regression, with the three-category frailty variable (robust/pre-frail/frail) as the outcome and robust as the reference category. OHRQoL and the number of remaining teeth were treated as continuous variables. Model 1 was a univariate model; Model 2 was adjusted for age and sex; and Model 3 was additionally adjusted for body mass index, education levels, and OHRQoL (in the teeth model) or the number of remaining teeth (in the OHRQoL model), smoking, alcohol drinking, diabetes mellitus, hypertension, and denture type.

**Table 1:** Demographics of participants with or without frailty.

Characteristic	All, *n* = 1,122	Robust, *n* = 521	Pre-frailty, *n* = 376	Frailty, *n* = 225	*P*-value
Sex					<0.01
Male, *n* (%)	441 (39.30)	234 (44.91)	124 (32.98)	83 (36.89)	
Female, *n* (%)	681 (60.70)	287 (55.09)	252 (67.02)	142 (63.11)	
Age, years, mean ± SD	77.97 ± 10.95	75.89 ± 10.21	78.12 ± 11.17	82.54 ± 10.85	<0.01
Education, *n* (%)					<0.01
Primary school and below, *n* (%)	324 (28.88)	118 (22.65)	113 (30.05)	93 (41.33)	
Junior high school, *n* (%)	483 (43.05)	238 (45.68)	172 (45.74)	73 (32.44)	
Senior high school and above, *n* (%)	315 (28.07)	165 (31.67)	91 (24.2)	59 (26.22)	
BMI (kg/m^2^), mean ± SD	23.15 ± 3.47	23.81 ± 3.69	22.76 ± 3.26	22.27 ± 2.97	<0.01
Cigarette smoking, *n* (%)	128 (11.41)	69 (13.24)	39 (10.37)	20 (8.89)	0.17
Alcohol drinking, *n* (%)	79 (7.04)	41 (7.87)	27 (7.18)	11 (4.89)	0.34
Edentulism, *n* (%)	196 (17.47)	57 (10.94)	69 (18.35)	70 (31.11)	<0.01
Remaining teeth (*n*), mean ± SD	14.14 ± 10.73	17.61 ± 10.44	12.83 ± 10.02	8.43 ± 9.56	<0.01
OHIP-14 score, mean ± SD	5.33 ± 8.59	2.31 ± 4.5	6.27 ± 8.64	10.76 ± 12.05	<0.01
Diabetes mellitus, *n* (%)	218 (19.43)	56 (10.75)	70 (18.62)	92 (40.89)	<0.01
Hypertension, *n* (%)	632 (56.33)	223 (42.80)	233 (61.97)	176 (78.22)	<0.01
Denture type, *n* (%)					<0.01
No denture	578 (51.52)	314 (60.27)	167 (44.41)	97 (43.11)	
Fixed crown/bridge	211 (18.81)	89 (17.08)	84 (22.34)	38 (16.89)	
Removable denture	333 (29.68)	118 (22.65)	125 (33.24)	90 (40.00)	

We used counterfactual-based causal mediation analysis to evaluate whether OHRQoL mediated the association between tooth loss (reflected as the number of remaining natural teeth) and frailty. The continuous mediator (OHRQoL) was modeled by linear regression and the three-category frailty outcome by multinomial logistic regression. We estimated the average causal mediation effect (indirect effect), the average direct effect, the total effect, and the proportion mediated (indirect/total × 100%) on the probability of each outcome category, with the probability of frailty as the primary effect. All mediation models were adjusted for age, sex, body mass index, education, smoking, alcohol drinking, diabetes mellitus, hypertension, and denture type. Confidence intervals (CIs) and *P*-values for all effects were obtained by non-parametric bootstrap with 5,000 resamples. We acknowledge that causal mediation analysis requires identifying assumptions (sequential ignorability) that cannot be fully verified in a cross-sectional study; the analysis is therefore interpreted as an exploratory decomposition of the cross-sectional association. As a sensitivity analysis, the mediation model was reestimated using an OHIP-14 score that excluded the functional limitation items (1–2), which most directly reflect tooth-loss consequences (Supplementary Table S1).

The reported estimates are log-odds coefficients derived from logistic regression models and are unstandardized coefficients. The *P*-values and 95% CIs were estimated in a two-tailed manner, and *P* < 0.05 was considered significant. Data were analyzed using SAS 9.4 (SAS Institute Inc., Cary, NC, USA).

## 3 Results

### 3.1 Baseline characteristics

Among the 1,122 participants, 225 were diagnosed with frailty, accounting for 20.05% of the participants. The mean age with standard deviation was 77.97 ± 10.95, and BMI was 23.15 ± 3.47. Women comprised 681 participants, making up 60.70% of the total. More than 70% of the participants had received junior high school education or below. Smokers made up 11.41% of the participants, with alcohol drinkers making up 7.04%. The average number of remaining teeth was 14.14, with an average OHIP-14 score of 5.33. Age, sex, education, BMI, number of remaining teeth, and OHIP-14 score were significantly different between participants with and without frailty (*P* < 0.05) (Table 1).

### 3.2 Association between number of remaining teeth and frailty

The ORs for number of remaining teeth among participants with or without frailty are presented in Table 2. Univariate Model 1 showed that number of remaining teeth was associated with 8.5% (OR = 0.915, 95% CI 0.899–0.930) lower odds of frailty. After controlling for sex and age, lower numbers of remaining teeth were significantly associated with frailty (OR = 0.923, 95% CI 0.907–0.940) (Model 2). Model 3 showed that number of remaining teeth was significantly associated with frailty, with OR = 0.922 (95% CI 0.903–0.942).

**Table 2:** OR (95% CI) for different stages of frailty by number of remaining teeth.

Stage of frailty	Model 1 OR (95% CI)	Model 2 OR (95% CI)	Model 3 OR (95% CI)
Robust	1.000 (reference)	1.000 (reference)	1.000 (reference)
Pre-frailty	0.957 (0.944–0.969)	0.960 (0.947–0.972)	0.968 (0.954–0.984)
Frailty	0.915 (0.899–0.930)	0.923 (0.907–0.940)	0.922 (0.903–0.942)

Model 1: univariate; Model 2: adjusted for sex and age; and Model 3: adjusted for age, sex, body mass index, education levels, OHRQoL, smoking, alcohol drinking, diabetes mellitus, hypertension, and denture type.

### 3.3 Association between OHRQoL and frailty

The ORs for OHRQoL among participants with or without frailty are presented in Table 3. Univariate Model 1 showed that OHRQoL was associated with a 15% (OR = 1.149, 95% CI 1.121–1.178) increase in the odds of frailty. After controlling age, sex, body mass index, education levels, number of remaining teeth, smoking, alcohol drinking, diabetes mellitus, hypertension, and denture type, Model 3 showed that OHRQoL was significantly associated with frailty, with OR = 1.140 (95% CI 1.109–1.171).

**Table 3:** OR (95% CI) for different stages of frailty by OHRQoL.

Stage of frailty	Model 1 OR (95% CI)	Model 2 OR (95% CI)	Model 3 OR (95% CI)
Robust	1.000 (reference)	1.000 (reference)	1.000 (reference)
Pre-frailty	1.102 (1.076–1.128)	1.106 (1.080–1.133)	1.090 (1.063–1.117)
Frailty	1.149 (1.121–1.178)	1.157 (1.128–1.188)	1.140 (1.109–1.171)

Model 1: univariate; Model 2: adjusted for sex and age; and Model 3: adjusted for age, sex, body mass index, education levels, number of remaining teeth, smoking, alcohol drinking, diabetes mellitus, hypertension, and denture type.

### 3.4 Mediation analysis of OHRQoL

Mediation analysis indicated that OHRQoL was statistically consistent with a potential partial mediating role in the association between remaining teeth and frailty (Table 4, Figure 1). We fitted a multinomial logistic outcome model for the three frailty categories (robust, pre-frail, and frail) and used counterfactual mediation analysis with 5,000 bootstrap resamples. For each additional remaining tooth, the estimated total effect on the probability of frailty was −0.0082 (95% CI, −0.0107 to −0.0055). The estimated indirect effect through OHRQoL was −0.0016 (95% CI, −0.0023 to −0.0010), and the estimated direct effect was −0.0067 (95% CI, −0.0091 to −0.0041). OHRQoL accounted for 19.3% (95% CI 11.6%–31.2%) of the association between remaining teeth and frailty. All mediation models were adjusted for age, sex, body mass index, education, smoking, alcohol drinking, diabetes mellitus, hypertension, and denture type. Reestimating the mediation model with an OHIP-14 score that excluded the functional limitation items (1–2) also yielded a materially unchanged proportion mediated of 17.2% (95% CI 10.1%–28.1%), supporting the robustness of the finding to the conceptual overlap between the exposure and the mediator.

**Table 4:** Mediation analysis of OHRQoL for the association between number of remaining teeth and frailty.

Effect	Estimate	95% CI	*P*-value
Number of remaining teeth → OHRQoL (mediator model, linear)	−0.1632	−0.2172 to −0.1093	<0.001
Indirect effect on probability of frailty	−0.0016	−0.0023 to −0.0010	<0.001
Direct effect on probability of frailty	−0.0067	−0.0091 to −0.0041	<0.001
Total effect on probability of frailty	−0.0082	−0.0107 to −0.0055	<0.001
Proportion mediated	19.3%	11.6% to 31.2%	<0.001

Three-category frailty outcome (robust/pre-frail/frail) modeled by multinomial logistic regression; counterfactual-based causal mediation with 5,000 bootstrap resamples. Effects are per one additional remaining natural tooth, on the probability of frailty. Adjusted for age, sex, body mass index, education, smoking, alcohol drinking, diabetes mellitus, hypertension, and denture type.

**Figure 1:**
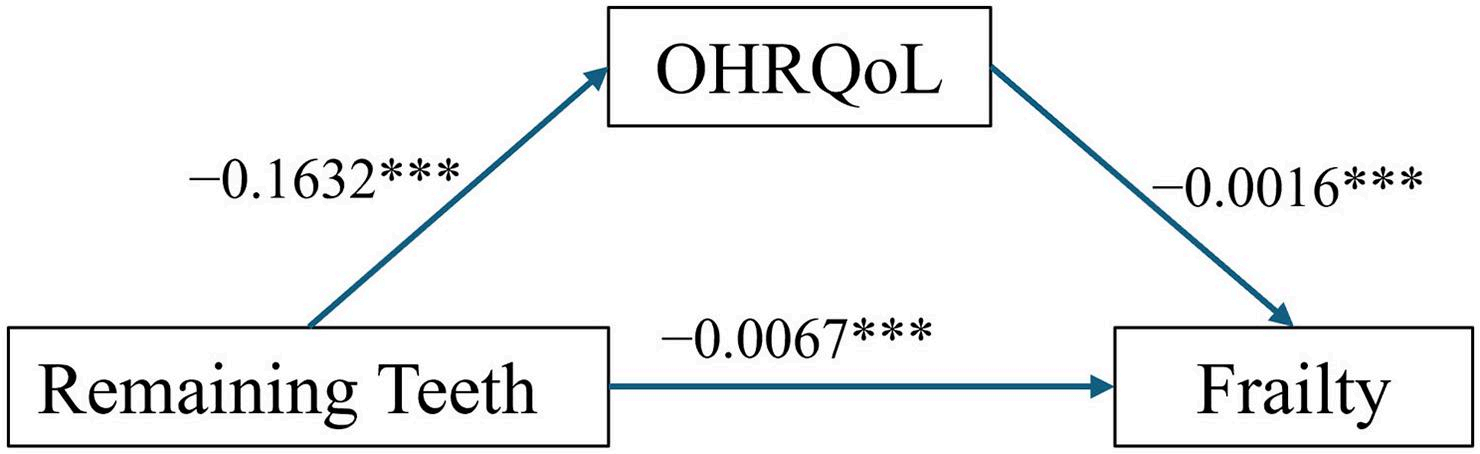
Mediation model of OHRQoL as a mediator of the association between remaining teeth and frailty; ** *p* < 0.01, *** *p* < 0.001.

## 4 Discussion

The present study demonstrates that the number of remaining teeth is significantly associated with frailty, and OHIP-14 may play a mediating role in this relationship.

A lower number of remaining teeth, indicating greater tooth loss, was associated with poorer OHRQoL as measured by the OHIP-14. This finding is consistent with behavioral, functional, and clinical links between tooth loss and OHRQoL; however, the cross-sectional design does not establish temporal order. Individuals with more tooth loss often experience persistent oral discomfort, impaired mastication, psychological distress, and social avoidance, which may be associated with reduced adherence to oral hygiene practices, delayed dental care-seeking, and under-treatment of early caries and periodontal disease. In a cross-sectional study conducted among 296 Indian adult dental patients aged 35–44 years, Veeraboina et al. adopted the validated OHIP-14 scale to evaluate OHRQoL and confirmed that the number and position of lost teeth exerted adverse effects on patients' OHRQoL, with an odds ratio of 6.7 (21). In longitudinal analyses, tooth loss has been shown to predict later poor OHRQoL, which may partly reflect compromised health behaviors and accelerated disease progression (22).

Multiple cohort and cross-sectional studies have suggested potential significant associations between frailty and oral health, consistent with our results. Dros et al. conducted a cross-sectional study among 170 older adults in the Netherlands and found that decreased oral health was associated with frailty (23). Tai et al. established risk prediction models for frailty based on the Taiwan Longitudinal Study on Aging survey and demonstrated that an increased OHIP-7T Q3 score (adj. OR = 1.33; 95% CI, 1.19–1.49) was significantly associated with frailty (24). Hakeem et al. reported a dose–response relationship between self-rated oral health and the frailty index using longitudinal data from the National Health and Nutrition and Examination Survey (25). Consistent with population-based longitudinal evidence, our study supports the hypothesis that tooth loss is associated with subjective oral health impairment. Meisel et al. conducted a 5- and 10-year longitudinal study and demonstrated that self-rated poor oral health was a strong and independent predictor of future tooth loss, with predictive power comparable to clinical periodontitis and caries examination. Our cross-sectional mediation model is consistent with a pathway in which tooth loss contributes to worse OHRQoL (22); the temporal order of these associations, however, cannot be confirmed given the cross-sectional design. Similarly, de Albuquerque et al. found that edentulism and untreated caries were strongly associated with lower OHRQoL in socioeconomically vulnerable populations (26). Kimmie-Dhansay et al. further noted that although tooth loss did not always reduce OHRQoL in socially normalized extraction populations, subjective dissatisfaction with oral health remained a strong predictor of impaired quality of life (27). In our study, lower numbers of remaining teeth were associated with both worse OHRQoL and higher frailty risk, consistent with these clinical and epidemiological observations. Our study extends previous findings by linking the number of remaining teeth, OHRQoL, and frailty. Gomes et al. demonstrated that premature tooth loss significantly impaired OHRQoL in children, highlighting the lifelong impact of tooth loss on subjective oral health (28).

There are several scales for assessing frailty. Previous studies have suggested that the FRAIL scale exhibits a relatively comparable sensitivity and specificity in predicting the risk of frailty to other frailty screening scales (29). The FRAIL scale can be accomplished with merely five self-assessment questions, presenting the advantage of being relatively simple and rapid (30). Previous research indicated that the prevalence of frailty among the elderly ranged from 26.8% to 62.8% (31–35). In a survey targeting 816 older adults in a Chinese community in Hong Kong, 12.5% of the participants had frailty assessed by the FRAIL scale, a lower prevalence than in our study, possibly due to the better economic conditions and medical services (36).

Age was identified as one of the most dominant and robust determinants of frailty in the present study, with frail participants being significantly older than non-frail participants, and age remaining statistically significant in all adjusted models. The findings were consistent with other studies (13–16, 19, 25, 30–33). This strong and independent effect of age suggests that aging-related processes may play a central role in the development of frailty. Tooth loss, poorer oral health-related quality of life, and frailty may partly represent parallel manifestations of biological aging and cumulative life course exposures. Therefore, the observed associations between OHRQoL, tooth loss, and frailty should be interpreted while considering the substantial influence of age. Although oral health indicators were significantly associated with frailty in this study, it remains possible that these factors may also serve as markers of cumulative aging.

The average number of remaining teeth among the participants was 14.14, with 196 elderly people being edentulous. However, Jiang et al. reported that the prevalence of edentulism among Chinese adults over 65 years old was about 5% (37), lower than the current study, which might be due to the higher average age of our participants. We observed that the two dimensions of OHIP-14 with the most serious impact on life were physical pain (1.77 ± 2.49) and functional limitation (1.14 ± 2.84). Shi-hua et al. found that elderly people over 60 years have higher scores in the two dimensions of physical pain and functional limitation (38), consistent with our results.

Our finding of the potential mediating role of OHRQoL in the association between number of remaining teeth and frailty is also plausible (Figure 1). Studies suggest that tooth loss/number of remaining teeth may be associated with reduced OHRQoL and subsequently with frailty. Kimble et al. conducted an 8-year cohort study involving 2,137 older adults and revealed that the greater the number of remaining teeth, the lower the risk of frailty (OR = 0.97, 95% CI: 0.95–1.00) (39). Hakeem et al. investigated 2,368 community-dwelling older adults in the United States from 2011 to 2014 and reached a similar conclusion, considering the number of remaining teeth as a protective factor against frailty (9).

We found that women had higher odds of frailty than men, which was consistent with the findings of Castrejón-Pérez et al. Women have a higher prevalence of frailty and faster aging speed than men (40). Elderly women are more prone to frailty, which may be due to the rapid decrease of estrogen in postmenopausal women, which has a negative impact on muscle and neuromuscular function (41). A cross-sectional study in Singapore also reported that elderly women had higher levels of interleukin-6 and C-reactive protein, which are also biomarkers of frailty (42).

In the present study, a significant association was found between educational level and frailty (Table 2), which was consistent with other studies. Some Chinese studies have reported that educational level may be a risk factor for frailty (43, 44), which might be due to age difference. Age is the primary risk factor for frailty, as the physical function of older adults gradually declines with age. This decline is characterized by reduced physiological reserve, weakened muscle strength, decreased tolerance to adverse events, and an increased risk of frailty (45, 46).

The current study suggests that low BMI in older adults is also significantly associated with frailty, consistent with previous findings (47). Low BMI is associated with frailty (48, 49), possibly due to the lower grip strength, unintentional weight loss, and reduced physical activity observed in older adults with low BMI (47). A study based on the 2001–2006 National Health and Nutrition Examination Survey (NHANES) in the United States indicated that a slight increase in BMI served as a protective factor against frailty and all-cause mortality (50). However, some studies have shown that increased BMI may be associated with frailty (51, 52). It has been hypothesized that this may result from the higher proportion of adipose tissue and a relative reduction in muscle mass in obese individuals, leading to decreased skeletal muscle strength and frailty caused by sarcopenia. In addition, older adults with higher BMI are at a greater risk of comorbidities such as diabetes, hypertension, and other chronic cardiovascular diseases, which may further contribute to frailty (53, 54). Some studies have also reported a U-shaped relationship between BMI and frailty, indicating that both low and high BMI are associated with an increased prevalence of frailty (55). The U-shaped relationship needs further research.

This study has several advantages and disadvantages. First, the present study applied exploratory mediation analysis in a cross-sectional design. Although mediation analysis was used to examine potential pathways, this study cannot confirm causal relationships or temporal order given its cross-sectional nature. Second, although we adjusted many confounders including BMI, chronic diseases, and denture type, some potentially relevant factors might still be missing. For example, we recorded denture type (no denture, fixed crown/bridge, removable complete or partial denture) and included it in the models, but we did not assess the functional fit of the prosthesis. The influence of tooth loss on the outcome might nonetheless be modified by the functional presence and fit of dentures. Third, detailed clinical dental data—including the cause of tooth loss, the distribution of missing teeth, the number of occluding tooth pairs, periodontal status, and the presence of caries—were not collected. Participants with a similar number of missing teeth may therefore have markedly different functional profiles. Fourth, the average age of the participants was nearly 80, with a prevalence of frailty of almost 20%, which is higher than most previous studies. The mean OHIP-14 score (5.33 ± 8.59) was relatively low, suggesting a generally good OHRQoL in the study population compared to some other cohorts. In addition, because participation required the ability to perform basic self-care and to communicate and complete the questionnaire, the most frail or cognitively impaired residents may have been underrepresented, potentially biasing the estimated associations toward the null and limiting generalizability to the frailest segment of the population. Our findings might not be generalized to other populations. Finally, OHIP-14 includes domains related to functional limitation, chewing difficulty, and discomfort, which are direct consequences of tooth loss. As such, there is potential conceptual and measurement overlap between the exposure (number of remaining teeth) and the proposed mediator (OHRQoL).

## 5 Conclusions

In conclusion, there might be a potential association between tooth loss and frailty, and OHRQoL may play a partial mediating role in this relationship. Oral health, particularly preventing tooth loss, should be a key focus for both the elderly and healthcare providers.

## Data Availability

This study suggests that OHRQoL is not only associated with frailty but may also act as a potential mediator, offering novel insights into possible pathways for intervention. The findings have practical implications for healthcare providers, emphasizing the importance of integrated care approaches that include oral health as a component of frailty management.

## Data availability statement

The datasets analyzed for this study are available from the corresponding author on reasonable request, subject to applicable ethical and participant-confidentiality restrictions.

## References

[1] World Health Organization. Ageing and Health. Geneva: World Health Organization (2024). Available online at: https://www.who.int/news-room/fact-sheets/detail/ageing-and-health (Accessed September 28, 2026).

[2] The Ministry of Civil Affairs of China, China National Working Commission on Ageing. The 2022 National Bulletin on Aging Affairs Development. Beijing: Ministry of Civil Affairs of the People's Republic of China and Office of the China National Committee on Ageing (2023). Available online at: https://www.gov.cn/lianbo/bumen/202312/content_6920261.htm (Accessed September 28, 2026).

[3] Hu Z Ma T. Comprehensive Analysis of the Impact of China’s Population Aging Trend. Beijing: Economic Forecasting Department of National Information Center (2023).

[4] Wang X. Report of the Fourth National Oral Health Epidemiological Survey. Beijing, China: People’s Medical Press (2018).

[5] Tu C Wang G Hu Z Wang S Yan Q Liu X. Burden of oral disorders, 1990–2019: estimates from the Global Burden of Disease Study 2019. Arch Med Sci. (2023) 19:930–40. 10.5114/aoms/165962 37560733 PMC10408023

[6] World Health Organization. Oral Health China 2022 Country profile. Geneva: World Health Organization. (2022). Available online at: https://www.who.int/publications/m/item/oral-health-chn-2022-country-profile (Accessed September 28, 2026).

[7] Bastos RS Sa LM Velasco SRM Teixeira DF Paino LS Vettore MV. Frailty and oral health-related quality of life in community-dwelling older adults: a cross-sectional study. Braz Oral Res. (2021) 35:e139. 10.1590/1807-3107bor-2021.vol35.0139 34932668

[8] Morinaga D Itoh T Soejima Y Horikawa T Nagai S Takeshita F. Relationships between test results for oral hypofunction, subjective frailty symptoms and oral health-related quality of life of Japanese dental outpatients: a multicentre, cross-sectional study. J Oral Rehabil. (2024) 51:2634–42. 10.1111/joor.13858 39305030

[9] Hakeem FF Bernabe E Sabbah W. Association between oral health and frailty among American older adults. J Am Med Dir Assoc. (2021) 22:559–+. 10.1016/j.jamda.2020.07.023 32859517

[10] dos Santos Nogueira DM de Matos Vieira AIC de Azevedo Reis E. Oral-motor function in older people: exploring new dimensions of frailty. J Oral Rehabil. (2023) 50:802–11. 10.1111/joor.13492 37162292

[11] Hong SW Baek J-H Kim K Kang J-H. Complex interplay of oral health, muscle and bone metabolism, and frailty in older individuals. Clin Oral Investig. (2024) 28:116. 10.1007/s00784-024-05521-9 38270679

[12] Xu X Zhao Y Wu B Pei Y Gu D. Association between tooth loss and frailty among Chinese older adults: the mediating role of dietary diversity. BMC Geriatr. (2023) 23:668. 10.1186/s12877-023-04355-6 37848821 PMC10583397

[13] Zhang J Xu G Xu L. Number of teeth and denture use are associated with frailty among Chinese older adults: a cohort study based on the CLHLS from 2008 to 2018. J. Nutr Health Aging. (2023) 27:960–5. 10.1007/s12603-023-2014-x 37997718 PMC12876819

[14] Wang M Deng X Chen H Diao Y Liu C Gao J. Frailty mediated the association between tooth loss and mortality in the oldest old individuals: a cohort study. Front Public Health. (2024) 11:1285226. 10.3389/fpubh.2023.1285226 38328540 PMC10848322

[15] Zhang Y Ge M Zhao W Hou L Xia X Liu X. Association between number of teeth, denture use and frailty: findings from the west China health and aging trend study. J Nutr Health Aging. (2020) 24:423–8. 10.1007/s12603-020-1346-z 32242210 PMC12876381

[16] Wibianty V Tadjoedin FM Lessang R Rizal MI Sulijaya B Natalina H. The association between periodontal status and frailty in elderly individuals. Open Dent J. (2024) 18:e18742106284116. 10.2174/0118742106284116240304035832

[17] Slade GD. Derivation and validation of a short-form oral health impact profile. Community Dent Oral Epidemiol. (1997) 25:284–90. 10.1111/j.1600-0528.1997.tb00941.x 9332805

[18] Xin W Ling J. Validation of a Chinese version of the oral health impact profile-14. Chin J Stomatol. (2006) 41(4):242–5. PMID: 16784595

[19] Abellan van Kan G Rolland Y Bergman H Morley JE Kritchevsky SB Vellas B. The I.A.N.A task force on frailty assessment of older people in clinical practice. J Nutr Health Aging. (2008) 12:29–37. 10.1007/BF02982161 18165842 PMC12892609

[20] Yin W Yanpei C Xiaoli Y Yan X. Localization and reliability and validity study of the FRAIL scale for screening frailty risk in elderly inpatients. Chin J Pract Nurs. (2018) 34:1526–30. 10.3760/cma.j.issn.1672-7088.2018.20.002

[21] Veeraboina N Doshi D Kulkarni S Patanapu SK Dantala SN Srilatha A. Tooth loss and oral health-related quality of life among adult dental patients: a cross-sectional study. Indian J Dent Res. (2022) 33:2–6. 10.4103/ijdr.IJDR_426_19 35946236

[22] Meisel P Holtfreter B Völzke H Kocher T. Self-reported oral health predicts tooth loss after five and ten years in a population-based study. J Clin Periodontol. (2018) 45:1164–72. 10.1111/jcpe.12997 30099763

[23] Dros C Sealy MJ Krijnen WP Weening-Verbree LF Hobbelen H Jager-Wittenaar H. Oral health and frailty in community-dwelling older adults in the northern Netherlands: a cross-sectional study. Int J Environ Res Public Health. (2022) 19:7654. 10.3390/ijerph19137654 35805314 PMC9265776

[24] Tai C-J Chen J-H Tseng T-G Lin Y-T Hsiao Y-H Lee M-C. Prediction of frailty and dementia using oral health impact profile from a population-based survey. Int J Environ Res Public Health. (2020) 17:1997. 10.3390/ijerph17061997 32197375 PMC7143751

[25] Hakeem FF Bernabe E Sabbah W. Self-rated oral health and frailty index among older Americans. Gerodontology. (2021) 38:185–90. 10.1111/ger.12513 33231321

[26] de Albuquerque LS de Queiroz RG Abanto J Strazzeri Bönecker MJ Soares Forte FD Sampaio FC. Dental caries, tooth loss and quality of life of individuals exposed to social risk factors in northeast Brazil. Int J Environ Res Public Health. (2023) 20:6661. 10.3390/ijerph20176661 37681801 PMC10487409

[27] Kimmie-Dhansay F Pontes CC Chikte UME Chinhenzva A Erasmus RT Kengne AP. Impacts of tooth loss on OHRQoL in an adult population in Cape Town, South Africa. Int J Environ Res Public Health. (2021) 18:4989. 10.3390/ijerph18094989 34066645 PMC8125865

[28] Gomes MC Perazzo MF Neves ÉTB Siqueira MBLD Paiva SM Granville-Garcia AF. Premature primary tooth loss and oral health-related quality of life in preschool children. Int J Environ Res Public Health. (2022) 19:12163. 10.3390/ijerph191912163 36231465 PMC9564822

[29] van Kan GA Rolland YM Morley JE Vellas B. Frailty: toward a clinical definition. J Am Med Dir Assoc. (2008) 9:71–2. 10.1016/j.jamda.2007.11.005 18261696

[30] Si H Jin Y Qiao X Tian X Liu X Wang C. Predictive performance of 7 frailty instruments for short-term disability, falls and hospitalization among Chinese community-dwelling older adults: a prospective cohort study. Int J Nurs Stud. (2021) 117:103875. 10.1016/j.ijnurstu.2021.103875 33621721

[31] He B Ma Y Wang C Jiang M Geng C Chang X. Prevalence and risk factors for frailty among community-dwelling older people in China: a systematic review and meta-analysis. J Nutr Health Aging. (2019) 23:442–50. 10.1007/s12603-019-1179-9 31021361 PMC12280368

[32] Collard RM Boter H Schoevers RA Oude Voshaar RC. Prevalence of frailty in community-dwelling older persons: a systematic review. J Am Geriatr Soc. (2012) 60:1487–92. 10.1111/j.1532-5415.2012.04054.x 22881367

[33] Akın S Mazıcıoglu MM Mucuk S Gocer S Deniz Şafak E Arguvanlı S. The prevalence of frailty and related factors in community-dwelling Turkish elderly according to modified fried frailty index and FRAIL scales. Aging Clin Exp Res. (2015) 27:703–9. 10.1007/s40520-015-0337-0 25762157

[34] Shamliyan T Talley KMC Ramakrishnan R Kane RL. Association of frailty with survival: a systematic literature review. Ageing Res Rev. (2013) 12:719–36. 10.1016/j.arr.2012.03.001 22426304

[35] King-Kallimanis BL Kenny RA Savva GM. Factor structure for the frailty syndrome was consistent across Europe. J Clin Epidemiol. (2014) 67:1008–15. 10.1016/j.jclinepi.2014.05.002 24948043

[36] Woo J Yu R Wong M Yeung F Wong M Lum C. Frailty screening in the community using the FRAIL scale. J Am Med Dir Assoc. (2015) 16:412–9. 10.1016/j.jamda.2015.01.087 25732832

[37] Jiang CM Chu CH Duangthip D Ettinger RL Hugo FN Kettratad-Pruksapong M. Global perspectives of oral health policies and oral healthcare schemes for older adult populations. Front Oral Health. (2021) 2:703526. 10.3389/froh.2021.703526 35048040 PMC8757822

[38] Shi-hua H Xianxing X Jing Z. Investigation of oral health related quality of life. Mil Med J South China. (2013) 27:153–5.

[39] Kimble R Papacosta AO Lennon LT Whincup PH Weyant RJ Mathers JC. The relationship of oral health with progression of physical frailty among older adults: a longitudinal study composed of two cohorts of older adults from the United Kingdom and United States. J Am Med Dir Assoc. (2023) 24:468–474.e3. 10.1016/j.jamda.2022.11.022 36584971 PMC10398566

[40] Castrejón-Pérez RC Borges-Yáñez SA Gutiérrez-Robledo LM Avila-Funes JA. Oral health conditions and frailty in Mexican community-dwelling elderly: a cross sectional analysis. BMC Public Health. (2012) 12:773. 10.1186/1471-2458-12-773 PMC3490998 22971075

[41] Jarach CM Tettamanti M Nobili A D’avanzo B. Social isolation and loneliness as related to progression and reversion of frailty in the survey of health aging retirement in Europe (SHARE). Age Ageing. (2021) 50:258–62. 10.1093/ageing/afaa168 32915990 PMC7793602

[42] Vaingankar JA Chong SA Abdin E Picco L Chua BY Shafie S. Prevalence of frailty and its association with sociodemographic and clinical characteristics, and resource utilization in a population of Singaporean older adults. Geriatr Gerontol Int. (2017) 17:1444–54. 10.1111/ggi.12891 27576598

[43] Ye R Yanfei G Shuangyuan S Zhezhou H Yang Z Yan S. Frailty status of population aged 50 and above in Shanghai. Chin J Disease Control. (2019) 23:445–51. 10.16462/j.cnki.zhjbkz.2019.04.016

[44] Jianhua H Chuan C Jiulin C Lihong P Haiping S. Prevalence of frailty and analysis of lifestyle-related factors in elderly residents of a suburban community in Shanghai. Shanghai Med J. (2021) 42:33–6.

[45] Fried LP Tangen CM Walston J Newman AB Hirsch C Gottdiener J. Frailty in older adults: evidence for a phenotype. J Gerontol A Biol Sci Med Sci. (2001) 56:M146–156. 10.1093/gerona/56.3.m146 11253156

[46] Li Q Zhenzhen L Libin G Liang C Yongyan H Qiang L. Factors influencing community frailty syndrome in elderly residents. Chin J Gen Pract. (2020) 23:598–603.

[47] Huynh TQH Pham TLA Vo VT Than HNT Nguyen TV. frailty and associated factors among the elderly in Vietnam: a cross-sectional study. Geriatrics (Basel). (2022) 7:85. 10.3390/geriatrics7040085 36005261 PMC9407721

[48] Soh Y Won CW. Sex differences in association between body composition and frailty or physical performance in community-dwelling older adults. Medicine (Baltimore). (2021) 100:e24400. 10.1097/MD.0000000000024400 33530237 PMC7850682

[49] São Romão Preto L Dias Conceição MdC Figueiredo TM Pereira Mata MA Barreira Preto PM Mateo Aguilar E. Frailty, body composition and nutritional status in non-institutionalised elderly. Enferm Clin. (2017) 27:339–45. 10.1016/j.enfcli.2017.06.004 28760658

[50] Jayanama K Theou O Godin J Cahill L Shivappa N Hébert JR. Relationship between diet quality scores and the risk of frailty and mortality in adults across a wide age spectrum. BMC Med. (2021) 19:64. 10.1186/s12916-021-01918-5 33722232 PMC7962372

[51] Kexin L. Analysis of the association between self-perceived aging and frailty in the elderly (Master’s thesis). Changchun University of Traditional Chinese Medicine (2023).

[52] Yanlin H Gui Q Feifan L. Prevalence and related factors of frailty syndrome in the elderly. Chin J Gerontol. (2023) 43:2270–3.

[53] Xu W Tan L Wang H-F Jiang T Tan M-S Tan L. Meta-analysis of modifiable risk factors for Alzheimer’s disease. J Neurol Neurosurg Psychiatry. (2015) 86:1299–306. 10.1136/jnnp-2015-310548 26294005

[54] Li X Xiong R. Association between frailty and gestational diabetes mellitus: a bidirectional and multivariable Mendelian randomization study. Front Endocrinol (Lausanne). (2024) 15:1382516. 10.3389/fendo.2024.1382516 38994009 PMC11236542

[55] Rietman ML van der A DL van Oostrom SH Picavet HSJ Dollé MET van Steeg H. The association between BMI and different frailty domains: a U-shaped curve? J Nutr Health Aging. (2018) 22:8–15. 10.1007/s12603-016-0854-3 29300416 PMC12880435

