# Supplementary material for: Number of remaining teeth and frailty in community-dwelling older adults: an exploratory analysis of the potential mediating role of oral health-related quality of life
Source: Front Oral Health. 2026 Oct 8;7:1927873. doi: 10.3389/froh.2026.1927873 (PMC13687001; doi:10.3389/froh.2026.1927873)
Supplement: Supplementary file 1 [file Table1.docx]

**Table S1 Sensitivity analysis of the mediation of OHRQoL for the association between tooth loss and frailty, excluding the OHIP-14 functional-limitation items (1–2)**

| **Effect** | **Primary analysis Estimate (95% CI)** | **Sensitivity analysis Estimate (95% CI)** |
| --- | --- | --- |
| Indirect effect (ACME) on probability of frailty | −0.0016 (−0.0023 to −0.0010) | −0.0014 (−0.0022 to −0.0008) |
| Direct effect (ADE) on probability of frailty | −0.0067 (−0.0091 to −0.0041) | −0.0068 (−0.0093 to −0.0043) |
| Total effect on probability of frailty | −0.0082 (−0.0107 to −0.0055) | −0.0083 (−0.0108 to −0.0056) |
| Proportion mediated | 19.3% (11.6% to 31.2%) | 17.2% (10.1% to 28.1%) |

*Note: Sensitivity analysis re-estimating the mediation model of Table 4 using an OHIP-14 score that excluded the functional-limitation items (1–2), the domain most directly reflective of tooth-loss consequences. Three-category frailty outcome (robust/pre-frail/frail) modeled by multinomial logistic regression; counterfactual-based causal mediation with 5,000 bootstrap resamples. Effects are per one additional remaining natural tooth, on the probability of frailty. Adjusted for the same covariates as in Table 4. P < 0.001 for all effects. n = 1,122 in both analyses.*
